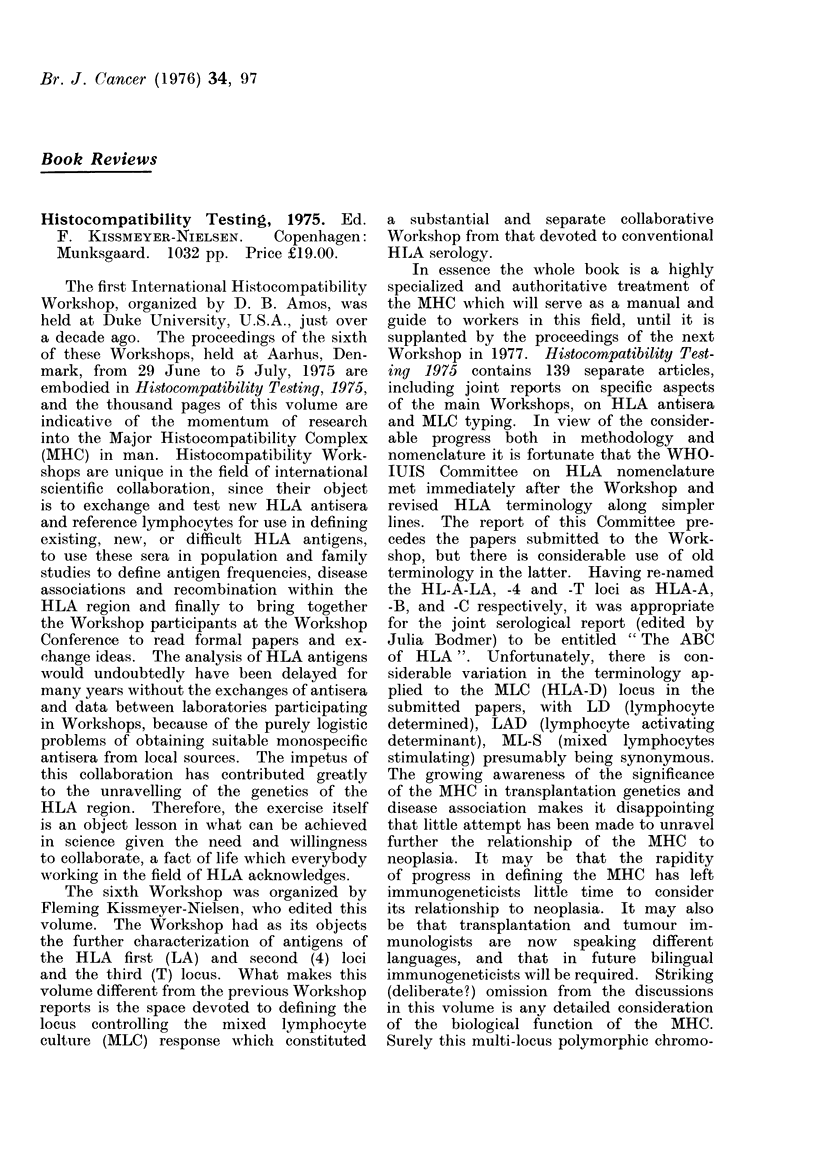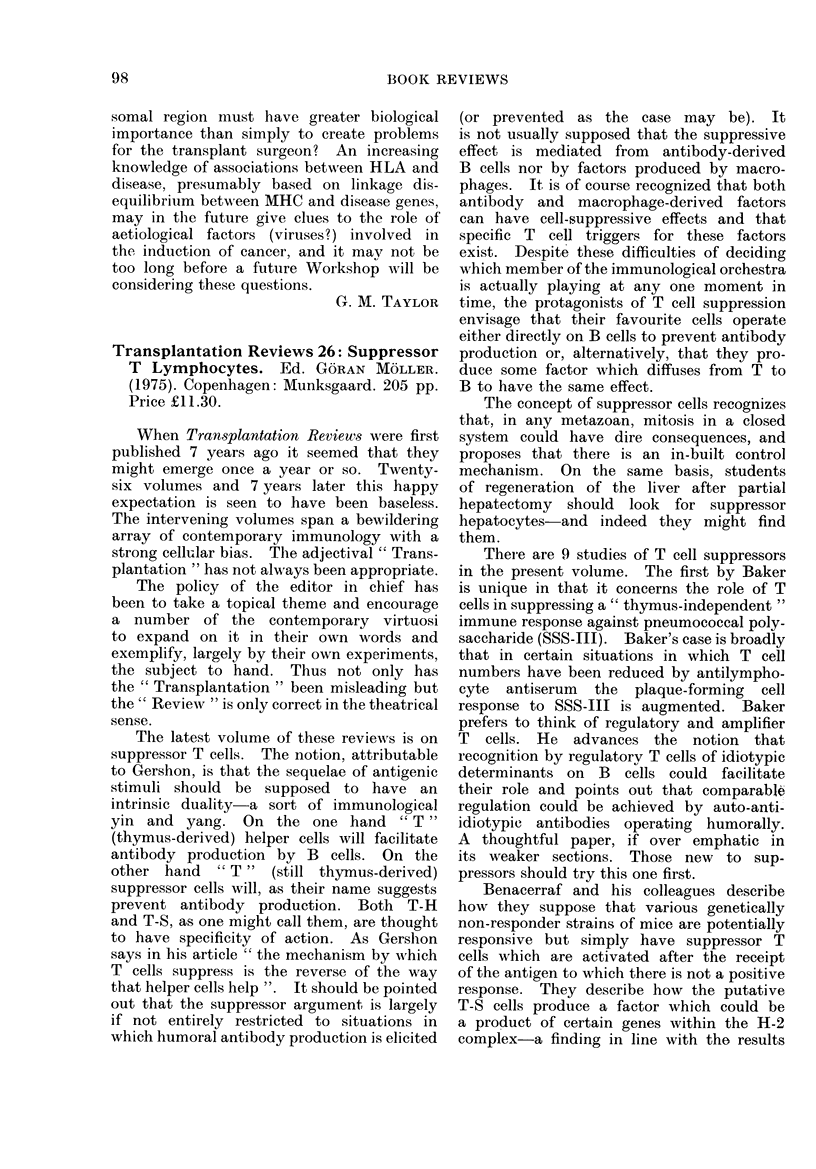# Histocompatibility Testing, 1975

**Published:** 1976-07

**Authors:** G. M. Taylor


*Br. J. Cancer* (1976) **34**, 97

### *Book Reviews*

**Histocompatibility Testing, 1975.** Ed. F. KISSMEYER-NIELSEN. Copenhagen: Munksgaard. 1032 pp. Price £19.00.

The first International Histocompatibility Workshop, organized by D. B. Amos, was held at Duke University, U.S.A., just over a decade ago. The proceedings of the sixth of these Workshops, held at Aarhus, Denmark, from 29 June to 5 July, 1975 are embodied in *Histocompatibility Testing, 1975*, and the thousand pages of this volume are indicative of the momentum of research into the Major Histocompatibility Complex (MHC) in man. Histocompatibility Workshops are unique in the field of international scientific collaboration, since their object is to exchange and test new HLA antisera and reference lymphocytes for use in defining existing, new, or difficult HLA antigens, to use these sera in population and family studies to define antigen frequencies, disease associations and recombination within the HLA region and finally to bring together the Workshop participants at the Workshop Conference to read formal papers and exchange ideas. The analysis of HLA antigens would undoubtedly have been delayed for many years without the exchanges of antisera and data between laboratories participating in Workshops, because of the purely logistic problems of obtaining suitable monospecific antisera from local sources. The impetus of this collaboration has contributed greatly to the unravelling of the genetics of the HLA region. Therefore, the exercise itself is an object lesson in what can be achieved in science given the need and willingness to collaborate, a fact of life which everybody working in the field of HLA acknowledges.

The sixth Workshop was organized by Fleming Kissmeyer-Nielsen, who edited this volume. The Workshop had as its objects the further characterization of antigens of the HLA first (LA) and second (4) loci and the third (T) locus. What makes this volume different from the previous Workshop reports is the space devoted to defining the locus controlling the mixed lymphocyte culture (MLC) response which constituted a substantial and separate collaborative Workshop from that devoted to conventional HLA serology.

In essence the whole book is a highly specialized and authoritative treatment of the MHC which will serve as a manual and guide to workers in this field, until it is supplanted by the proceedings of the next Workshop in 1977. *Histocompatibility Testing 1975* contains 139 separate articles, including joint reports on specific aspects of the main Workshops, on HLA antisera and MLC typing. In view of the considerable progress both in methodology and nomenclature it is fortunate that the WHO-IUIS Committee on HLA nomenclature met immediately after the Workshop and revised HLA terminology along simpler lines. The report of this Committee precedes the papers submitted to the Workshop, but there is considerable use of old terminology in the latter. Having re-named the HL-A-LA, -4 and -T loci as HLA-A, -B, and -C respectively, it was appropriate for the joint serological report (edited by Julia Bodmer) to be entitled "The ABC of HLA". Unfortunately, there is considerable variation in the terminology applied to the MLC (HLA-D) locus in the submitted papers, with LD (lymphocyte determined), LAD (lymphocyte activating determinant), ML-S (mixed lymphocytes stimulating) presumably being synonymous. The growing awareness of the significance of the MHC in transplantation genetics and disease association makes it disappointing that little attempt has been made to unravel further the relationship of the MHC to neoplasia. It may be that the rapidity of progress in defining the MHC has left immunogeneticists little time to consider its relationship to neoplasia. It may also be that transplantation and tumour immunologists are now speaking different languages, and that in future bilingual immunogeneticists will be required. Striking (deliberate?) omission from the discussions in this volume is any detailed consideration of the biological function of the MHC. Surely this multi-locus polymorphic chromosomal region must have greater biological importance than simply to create problems for the transplant surgeon? An increasing knowledge of associations between HLA and disease, presumably based on linkage disequilibrium between MHC and disease genes, may in the future give clues to the role of aetiological factors (viruses?) involved in the induction of cancer, and it may not be too long before a future Workshop will be considering these questions.

G. M. TAYLOR